# Study on the Microstructure and Mechanical Properties of 7085 Aluminum Alloy Reinforced by In Situ (ZrB_2_ + Al_2_O_3_) Nanoparticles and Rare Earth Er

**DOI:** 10.3390/ma18092009

**Published:** 2025-04-29

**Authors:** Yuqiang Zhang, Yutao Zhao, Xizhou Kai, Jiadong Yang, Hanfei Zhu, Ying Shan

**Affiliations:** School of Material Science and Engineering, Jiangsu University, Zhenjiang 212013, China; 18252929540@163.com (Y.Z.); kaixizhou@ujs.edu.cn (X.K.); yjd1930299617@163.com (J.Y.); zhf18012849965@163.com (H.Z.); 15716100691@163.com (Y.S.)

**Keywords:** ZrB_2_ and Al_2_O_3_ nanoparticles, 7085 Al alloy, rare earth Er, microstructure, mechanical properties

## Abstract

This study investigates the synergistic strengthening effects of in situ synthesized nano (ZrB_2_ + Al_2_O_3_) particles and rare earth Er microalloying on the microstructure and mechanical properties of 7085 aluminum alloy. The composite material was prepared through a melt direct reaction combined with rolling and T6 heat treatment, with microstructural evolution characterized by metallurgical microscopy, XRD, and SEM. Results demonstrate that the addition of 3 vol.% in situ nano (ZrB_2_ + Al_2_O_3_) particles optimally enhances both strength and toughness, achieving a tensile strength of 635.4 MPa (16.2% increase) and elongation after fracture of 16.2% (14.9% improvement) compared to the matrix alloy. Excessive particle content (5 vol.%) leads to severe clustering and deteriorated interfacial bonding, causing performance degradation. Introducing 0.3 wt.% Er improves particle distribution uniformity and promotes Al_3_(Er,Zr) precipitate formation, refining grains and strengthening interfaces. This further elevates tensile strength to 654.8 MPa (19.7% increase) and elongation to 16.6% (17.7% improvement). The research reveals the synergistic optimization mechanism between particle content and Er addition, providing theoretical support for designing high-performance aluminum matrix composites.

## 1. Introduction

Al-Zn-Mg-Cu aluminum alloys exhibit high strength, excellent toughness, good thermal resistance, corrosion resistance, and processability, making them widely applicable across various fields [1,2,3]. The 3xxx (Al-Mn series), 5xxx (Al-Mg series), 6xxx (Al-Mg-Si series), and 7xxx (Al-Zn-Mg-Cu series) aluminum alloys have undergone distinct advancements in aerospace applications to overcome their inherent limitations [4,5]. The 3xxx alloys (such as AA3003) employ laminate compositing and cold deformation techniques (such as AA3003/AA4043 hybrid materials) to achieve tensile strengths of up to 280 MPa. However, their relatively low strength confines their use to non-load-bearing components like fuel tanks. 5xxx alloys (such as AA5083) leverage micro-alloying (with Sc or Zr) and superplastic-forming technology to enhance strength to 400 MPa while mitigating stress corrosion cracking. These alloys are widely adopted in cryogenic fuel storage tanks for spacecraft due to their corrosion resistance and stability at low temperatures. The 6xxx alloys (such as AA6061) utilize precipitation hardening (via T6/T7 aging) and particle-reinforced composites (such as SiC/Al_2_O_3_) to reach strengths of 450 MPa. Despite these improvements, their susceptibility to thermal softening above 150 °C and limited ductility restrict their application to secondary structures like door rails. In contrast, 7xxx alloys (such as AA7075, AA7085) have emerged as the premier choice for extreme-load components such as aircraft wing spars and landing gear. Through nanocomposite reinforcement (such as SiC/graphene), in situ synthesis of dispersed nanoparticles (such as ZrB_2_/TiB_2_), and rare-earth micro-alloying (such as Er/Sc additions), these alloys achieve ultrahigh tensile strengths exceeding 650 MPa while maintaining damage tolerance. Their exceptional specific strength (250 MPa·cm^3^/g) surpasses other series, and advanced over-aging treatments coupled with interfacial optimization resolve historical challenges like stress corrosion susceptibility and brittleness. These innovations align with aerospace demands for lightweight design, operational reliability, and extended service life, solidifying the 7xxx series’ dominance in critical aerospace applications.

This study focuses on the 7085 aluminum alloy, a high-zinc variant within the Al-Zn-Mg-Cu series. As a member of the Al-Zn-Mg-Cu alloy family, the 7085 aluminum alloy is specifically designed for high-strength applications. It combines exceptional strength and toughness with favorable machinability and weldability, enabling its extensive use in aerospace engineering [6,7]. As aerospace technologies advance toward lightweight design, enhanced reliability, and extended service durability, conventional 7085 aluminum alloy (ultimate tensile strength~550 MPa, elongation 12–15%) [8] increasingly fails to satisfy the stringent performance demands of next-generation aircraft for mission-critical structural components, particularly wing spars and landing gear support assemblies. A representative case involves commercial aircraft primary load-bearing frames [9], which require sustained tensile strength exceeding 600 MPa coupled with fracture toughness greater than 30 MPa·m^1^/^2^ when subjected to cyclic temperature variations between −60 °C and 150 °C. Current 7085 alloys exhibit notable shortcomings in fatigue resistance and damage tolerance under multi-axial loading scenarios. Furthermore, the inherent strength–ductility trade-off phenomenon in aluminum alloys frequently results in compromised fracture toughness during strength optimization, raising concerns about catastrophic brittle failure mechanisms. Consequently, the development of an advanced 7085 aluminum alloy variant that synergistically combines ultrahigh tensile strength (>650 MPa), enhanced ductility (>15% elongation), and superior fracture toughness has emerged as a critical research priority for overcoming existing material limitations in aerospace engineering applications. In the context of increasingly stringent performance requirements for aircraft materials [10], developing cost-effective methods to manufacture aluminum alloys with higher strength grades has become a key research focus in this field, offering vast potential for future advancements.

Currently, to address the aforementioned challenges, numerous scholars have conducted extensive research, primarily adopting the following methods: (1) Ceramic particle reinforcement [11,12]. Ceramic-particle-reinforced aluminum matrix composites significantly enhance mechanical properties by uniformly dispersing high-hardness, high-stiffness ceramic particles (such as SiC and Al_2_O_3_) into the aluminum matrix. As reinforcing phases, ceramic particles effectively transfer loads and hinder matrix deformation, thereby improving the overall strength and elastic modulus of the material. Simultaneously, interfacial interactions between particles and the matrix suppress dislocation motion, enhancing creep and fatigue resistance. Under high-temperature conditions, ceramic particles stabilize the matrix structure, delaying the softening tendency of the aluminum alloy and enabling the material to retain elevated strength at elevated temperatures. Additionally, the dispersion strengthening effect and grain refinement strengthening mechanism further optimize the material’s hardness and wear resistance, enabling excellent performance under friction or impact loads. (2) Rare earth microalloying [13,14]. Rare earth microalloying-enhanced aluminum matrix composites are advanced materials that leverage the unique physicochemical properties of trace rare earth elements (such as Sc, Ce, La, and Y) to optimize microstructures and interfacial configurations, thereby significantly improving mechanical properties. The addition of rare earth elements effectively refines matrix grains and purifies the melt, reducing the size and distribution of impurity phases. These elements also interact with the aluminum matrix or reinforcing phases (such as ceramic particles), forming highly stable rare earth compounds or interfacial transition layers to enhance interfacial bonding strength. This microalloying effect synergistically boosts strength, hardness, and creep resistance through mechanisms such as grain refinement strengthening, solid solution strengthening, and secondary phase strengthening, while mitigating the toughness reduction caused by the introduction of reinforcing phases in traditional aluminum matrix composites. Furthermore, rare earth elements inhibit high-temperature grain boundary sliding and dislocation motion, ensuring better mechanical stability in high-temperature environments.

Ceramic particle reinforcement is a type of exogenous particle strengthening. This approach suffers from drawbacks such as poor wettability, uncontrollable interfacial reactions, and uneven particle distribution [15,16]. This study employs in situ synthesized nanoparticle-reinforced aluminum matrix composites [17,18], where the reinforcing phase is generated through in situ chemical reactions within the aluminum matrix. “In situ” refers to the phenomenon where nanoparticles directly nucleate and grow within the matrix during the material preparation process (such as the melting stage), rather than being externally added through methods like pre-synthesis followed by mechanical mixing. This synthesis strategy ensures that the reinforcing particles possess ultra-clean surfaces, and the interfacial bonding regions between the reinforcement and matrix exhibit no defects or pores, significantly enhancing the interfacial bonding quality. Such in situ self-generated characteristics effectively circumvent the common issues of interfacial contamination and structural discontinuity associated with traditional exogenous particle addition methods. ZrB_2_ particles possess an ultra-high melting point (3250 °C) and excellent high-temperature stability. Through in situ synthesis, nanoscale-dispersed ZrB_2_ can be generated within the aluminum matrix. Their low lattice mismatch with the aluminum matrix ensures clean interfacial bonding, significantly enhancing the alloy’s strength. Al_2_O_3_ particles leverage their high hardness (~20 GPa) and chemical inertness to hinder dislocation motion via the Orowan strengthening mechanism, while simultaneously improving the composite’s wear resistance and toughness. The controlled differences in thermal expansion coefficients between both particles and the aluminum matrix effectively reduce interfacial thermal stress, preventing crack initiation. Furthermore, the synergistic effect of ZrB_2_/Al_2_O_3_ balances the strength–ductility trade-off; ZrB_2_ dominates high-temperature strengthening, while Al_2_O_3_ optimizes room-temperature toughness, making the composite suitable for extreme aerospace operating conditions. For instance, Vineet K et al. [19] utilized a mixed-salt method to in situ synthesize ZrB_2_ particles in ZA alloys by adding KBF_4_ and KZrF_6_. The incorporation of ZrB_2_ particles refined the matrix grains, significantly enhancing the mechanical properties of the alloy. Compared to the ZA alloy, the ZA/9 vol.% ZrB_2_ composite exhibited a 34% increase in tensile strength, 40% improvement in compressive strength, and 62% higher hardness. Similarly, Zhao et al. [20] synthesized TiB_2_ particles in situ in a 6201 aluminum alloy using K_2_TiF_6_ and KBF_4_. Their study revealed two types of TiB_2_ particles: submicron-sized particles forming a network distribution and nano-sized dispersoids. The 4 wt.% TiB_2_/6201Al composite achieved ultimate tensile strength (UTS) of 360.9 MPa, elongation (El) of 8.27%, and electrical conductivity (EC) of 53.5% IACS. The semi-coherent particle/matrix (p/m) interface formed in the composite played a critical role in strengthening the matrix.

Single particle reinforcement or single rare earth microalloying [21,22,23] provides limited optimization for the performance of aluminum matrix composites. Therefore, this experiment combines rare earth microalloying with nanoparticle reinforcement to better enhance the mechanical properties of aluminum alloys. This study uses the rare earth element Er to enhance the mechanical properties of aluminum alloys. Although scandium (Sc) exhibits the best strengthening effect in aluminum matrix composites [24], its high cost limits practical applications. Er, belonging to the same group as Sc, offers similar grain refinement and second-phase strengthening capabilities at a significantly lower price, making it a cost-effective alternative. Er improves material strength and wear resistance by forming reinforcing phases that pin dislocations, refining the microstructure and purifying the melt to enhance interfacial bonding. Importantly, it achieves a synergistic optimization of strength and ductility with minimal adverse effects on toughness. For example, Li et al. [25] demonstrated that adding rare earth Er to 7075 aluminum alloys promoted the formation of coherent Al_3_Er precipitates with the Al matrix. This resulted in grain refinement, reducing the length of columnar crystals from 106 mm to 84 mm, and achieving a tensile strength of 338 MPa with excellent plasticity (15.9% elongation). Similarly, Qian et al. [26] investigated the addition of rare earth Er and ZrB_2_ particles to 6061 aluminum alloys. They observed a 2 nm-thick Er-rich layer coating the surface of ZrB_2_ particles, which effectively mitigated particle agglomeration and significantly enhanced the composite’s overall performance. Huang et al. [27] found that adding Er to 7055 aluminum alloy forms thermally stable Al_3_(Er,Zr) precipitates. These precipitates optimize interfacial structures and refine grains. Combined with Al_2_O_3_ and ZrB_2_ nanoparticles, Er enables multi-scale strengthening, markedly improving the alloy’s high-temperature creep resistance and microstructural stability. Kim et al. [28] demonstrated that Er addition to Al-Zn-Mg alloys synergistically with Zr induces L12-structured Al_3_(Zr,Er) precipitates with a core–shell configuration (Er-rich core/Zr-rich shell). The diffusion-resistant Zr-rich shell effectively stabilizes them against coarsening at elevated temperatures. In Zr-Er-Y alloys, Er boosts L12 phase density by ~20% compared to Zr/Y mono-doped systems, facilitating heterogeneous nucleation of the η-phase and leading to refined, dispersed η-phase precipitation during aging.

This study introduces an innovative approach combining in situ nanoparticle synthesis with Er microalloying to overcome traditional limitations in aluminum composite design. Unlike previous methods that relied on single reinforcement techniques (such as Vineet K et al. [19], Zhao et al. [20]) or costly Sc alloying, our strategy synergistically integrates hybrid ZrB_2_-Al_2_O_3_ nanoparticle formation and Er modification. The in situ process eliminates interfacial defects common in conventional composites, while Er simultaneously refines grain structure and stabilizes nanoscale precipitates through thermally stable core–shell phases. This multi-scale reinforcement mechanism achieves an unprecedented balance between strength and ductility, addressing the typical trade-offs in particle-reinforced systems and rare-earth-modified alloys. By harmonizing nanoparticle dispersion, precipitation hardening, and microstructural control, the work offers a new pathway for developing high-performance aluminum composites with enhanced reliability for aerospace applications.

## 2. Materials and Methods

### 2.1. Selection of Matrix Material and Reinforcement Phase

The 7085 aluminum alloy was selected as the matrix material in this experiment, with its primary chemical composition listed in Table 1. Quantitative elemental analysis was conducted using a mass spectrometer; the mass fractions of Fe, Si, Mn, Cr, and Ti were all below 0.1% and thus considered negligible in this study. The raw materials included commercially pure aluminum (Al), zinc (Zn), copper (Cu), magnesium (Mg), as well as Al-10Zr and Al-20Er master alloys. The Na_2_B_4_O_7_-K_2_ZrF_6_ reaction system was employed to in situ synthesize ZrB_2_ and Al_2_O_3_ particles within the matrix.

### 2.2. Fabrication of Composite Materials

In this experiment, 7085 aluminum matrix composites synergistically reinforced with ZrB_2_, Al_2_O_3_ particles, and Er were prepared via the melt direct reaction method. The detailed procedure is as follows: First, industrial-grade Na_2_B_4_O_7_ and K_2_ZrF_6_ were dried; in addition to synthesizing particles in situ with NaB_4_O_7_, K_2_ZrF_6_ reacts with the aluminum oxide layer upon melting to generate fluorides, disrupting the alumina film that hinders wetting, thereby improving the wettability of the aluminum/ceramic interface [29]. Borax and zirconium salt were placed in an oven and dried at 200 °C for 3 h. After drying, they were ground into powder in a mortar, mixed uniformly, and wrapped in aluminum foil for later use. Next, commercially pure aluminum was melted in an electromagnetic induction furnace at 850 °C. The pre-mixed borax and zirconium salt powders were added batchwise into the molten aluminum using a graphite bell jar, followed by continuous stirring with a graphite rod to prevent agglomeration. The reaction proceeded at 850 °C for 30 min, and electromagnetic stirring (200 A, 10 Hz) also continued for 30 min in this process. After the reaction, the melt was degassed and slag-removed. When the melt temperature dropped to 750 °C, commercially pure copper, zinc, Al-10Zr, and Al-20Er (if only in situ particles are required in the experiment, this step can be omitted) master alloys were added, and electromagnetic stirring continued for 20 min in this process. After 20 min, when the temperature decreased to 700 °C, commercially pure magnesium was introduced and reacted for 10 min; electromagnetic stirring also continued for 10 min in this process. The melt was degassed and slag-removed (by-product removal) again. Finally, at 720 °C, the melt was poured into preheated molds and cooled to obtain composite ingots.

Considering that the thermal expansion of different particles or rare earth elements differs from that of the 7085 aluminum alloy [30], during T6 heat treatment, different parameters should be adopted to ensure better heat treatment effects for the samples. For the base alloy without any additives, the solution treatment temperature is 470 °C, which is a conventional single-stage process. The holding time is 2 h to ensure sufficient dissolution of Cu, Zn, and Mg elements. The heating rate should not exceed 10 °C per minute to avoid local overheating and grain coarsening. Water quenching is conducted after holding is completed. Artificial aging is performed at 120 °C for 24 h.

For the 7085 aluminum alloy reinforced with ZrB_2_ and Al_2_O_3_ particles, the thermal expansion coefficient difference between the particles and the aluminum alloy induces dislocation multiplication effects. A higher solution temperature and longer solution time are required to activate interfacial bonding. Thus, the solution treatment is conducted at 520 °C for 4 h. Water quenching is conducted after holding is completed. Artificial aging is extended to 120 °C for 28 h to compensate for the diffusion hindrance caused by the particles.

For the 7085 aluminum alloy co-reinforced with ZrB_2_ and Al_2_O_3_ particles and rare earth Er, a higher solution treatment temperature is necessary to activate interfacial reactions. Due to the higher diffusion coefficient of Er, the aging temperature must be reduced to suppress the coarsening of Er-containing phases. A multi-stage solution treatment optimizes microstructural homogeneity. The solution treatment follows a three-stage process: 500 °C for 2 h, then 520 °C for 2 h, followed by 540 °C for 2 h. Water quenching is conducted after holding is completed. Artificial aging is performed at 100 °C for 30 h.

### 2.3. Rolling Process of Composites

The as-cast 7085 aluminum matrix composite was cut into rectangular specimens (60 × 30 × 12 mm^3^). To reduce segregation, the specimens underwent homogenization treatment at 470 °C for 24 h in a furnace. After homogenization, all six surfaces of the specimens were polished with sandpaper, degreased in acetone, and rinsed with alcohol.

Before rolling, the rollers were lubricated and preheated. The initial roll gap was set to 12 mm. Specimens preheated in a muffle furnace at 470 °C were rolled with a 2 mm reduction per pass. After each pass, the specimens were reheated at 470 °C for 10 min. This process was repeated until the final thickness reached 2 mm.

### 2.4. Microstructural Characterization and Mechanical Testing

Metallographic specimens of the 7085 aluminum alloy were mechanically ground and polished using 240~2000# sandpaper, followed by rough and fine polishing. The Zeiss Observer Z1m optical microscope (Carl Zeiss AG, Oberkochen, Germany) was employed to observe the microstructure, including grain size, morphology, and the size/distribution of particle clusters. Grain size was measured using Image-J software( the version number: 1.54g) by outlining all grains in the acquired micrographs, yielding an average value with a measurement error of ±5%. The primary source of measurement error stems from inaccuracies introduced during manual grain boundary delineation. Phase composition was determined using a D8 ADVANCE X-ray diffractometer (Bruker AXS GmbH, Karlsruhe, Germany). The FEI NovaNano450 scanning electron microscope (SEM) (FEI Company, Hillsboro, OR, USA) was utilized to characterize the size, distribution, and morphology of particle clusters, as well as the dispersion of precipitates in the composites. The H-7800 transmission electron microscope (TEM) (Hitachi High-Technologies Corporation, Tokyo, Japan) was applied to analyze dislocation distribution and the interfacial relationships between particles/precipitates and the aluminum matrix. Room-temperature tensile tests were conducted on an AG-X Plus precision universal testing machine (Shimadzu Corporation, Tokyo, Japan) at a strain rate of 1 mm/min, and the test results were averaged from three specimens. The UTS values presented have a measurement error of ±3%, primarily caused by the load cell accuracy of the testing machine, specimen misalignment, and strain rate fluctuations. The elongation measurement error of ±3% is primarily attributed to extensometer grip slippage and calibration deviations.

## 3. Results

### 3.1. Microstructure of (ZrB_2_ + Al_2_O_3_)/7085 Composites

**Figure 1:**
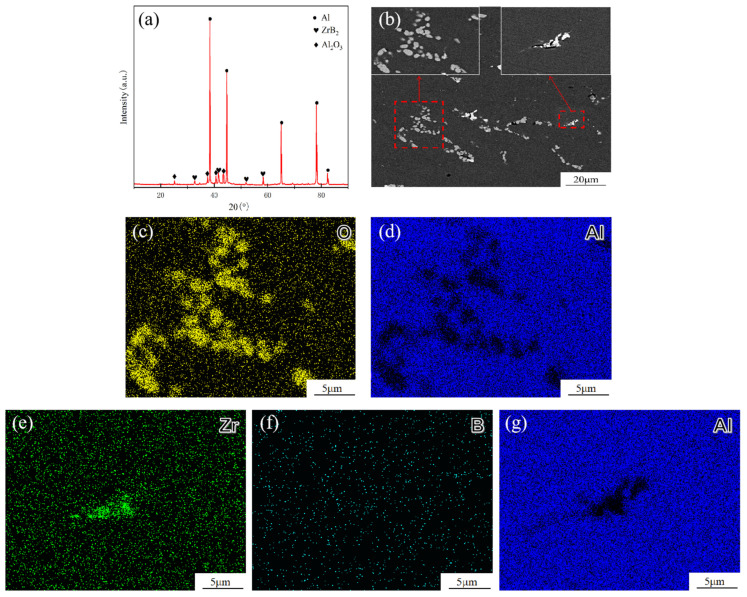
ZrB_2_, Al_2_O_3_/7085Al composites: (**a**) XRD; (**b**) SEM; (**c**–**g**) EDS mapping analysis of O, Zr, B, and Al elements.

Figure 1 shows the X-ray diffraction (XRD) patterns, SEM micrographs, and EDS elemental mapping of the in situ ZrB_2_ and Al_2_O_3_ nanoparticle-reinforced 7085Al composite. From the XRD pattern, diffraction peaks corresponding to Al, ZrB_2_, and Al_2_O_3_ are observed, confirming the successful formation of ZrB_2_ and Al_2_O_3_ particles in the 7085Al composite. The EDS elemental mapping (Figure 1c–d) reveals that the clusters are composed of Al and O elements, while the clusters in Figure 1e–g consist of Zr and B elements. Notably, the B signal appears weak in the EDS maps due to its low atomic weight. Combined with XRD and EDS analyses, these results confirm the successful in situ synthesis of ZrB_2_ and Al_2_O_3_ particles in the 7085Al composite. The metallurgical reaction for the in situ formation of ZrB_2_ particles via the reaction between potassium fluorozirconate (K_2_ZrF_6_) and sodium tetraborate ((Na_2_B_4_O_7_·10H_2_O) is as follows:

(1) $$9NaB_{4}O_{7}+30K_{2}ZrF_{6}+60Al=12ZrO_{2}+18ZrB_{2}+13{Al}_{2}O_{3}+18K_{2}NaAlF_{6}+16AlF_{3}+24KF$$

Based on the calculation using Equation (1), the volume ratio of ZrB_2_ to Al_2_O_3_ particles is determined to be 0.96:1. The K_2_NaAlF_6_, AlF_3_, and KF in the products are typical molten salt by-products formed during high-temperature reactions, which are removed through subsequent slag removal treatment.

**Figure 2:**
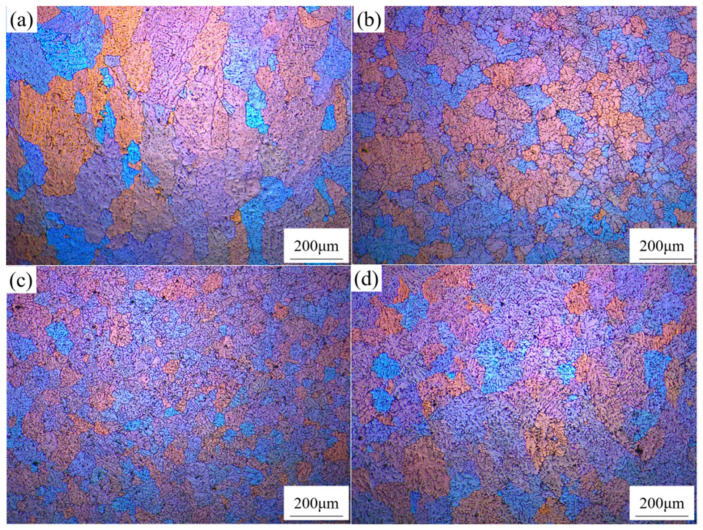
OM patterns of 7085Al composites reinforced by ZrB_2_, Al_2_O_3_ nanoparticles with different contents: (**a**) 0 vol.% (ZrB_2_ + Al_2_O_3_); (**b**) 1 vol.% (ZrB_2_ + Al_2_O_3_); (**c**) 3 vol.% (ZrB_2_ + Al_2_O_3_); (**d**) 5 vol.% (ZrB_2_ + Al_2_O_3_).

Figure 2 shows polarized light micrographs of 7085Al composites reinforced with varying contents of ZrB_2_ and Al_2_O_3_ particles. In Figure 2a, where no nanoparticles are added, the grains are relatively coarse, with an average grain size of 121.3 μm. In Figure 2b, with the addition of 1 vol.% nanoparticles, the grains are refined, yielding an average grain size of 53.8 μm. In Figure 2c, with 3 vol.% nanoparticles, further refinement occurs, resulting in an average grain size of 40.6 μm. In Figure 2d, with 5 vol.% nanoparticles, grain coarsening is observed instead of refinement, and the average grain size increases to 63.8 μm. Grain refinement is attributed to two mechanisms: (1) heterogeneous nucleation: ZrB_2_ and Al_2_O_3_ particles act as nucleation sites, promoting grain formation and increasing grain density, thereby refining the microstructure; (2) grain boundary pinning: the particles predominantly distribute at grain boundaries, inhibiting grain boundary migration and suppressing grain growth. At 5 vol.% particle content, severe agglomeration occurs at grain boundaries, diminishing the inhibitory effect on grain boundary migration and leading to abnormal grain coarsening.

**Figure 3:**
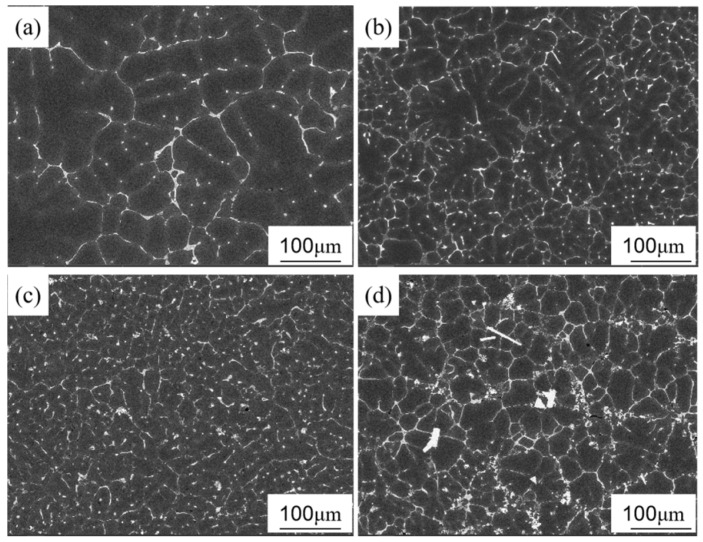
SEM micrographs of 7085Al composites reinforced by ZrB_2_, Al_2_O_3_ nanoparticles with different contents: (**a**) 0 vol.% (ZrB_2_ + Al_2_O_3_); (**b**) 1 vol.% (ZrB_2_ + Al_2_O_3_); (**c**) 3 vol.% (ZrB_2_ + Al_2_O_3_); (**d**) 5 vol.% (ZrB_2_ + Al_2_O_3_).

Figure 3 shows SEM images of 7085Al composites reinforced with varying contents of ZrB_2_ and Al_2_O_3_ particles. As shown in Figure 3a, when no particles are added, the grains are relatively coarse, which is attributed to continuous undercooling during the solidification of the alloy [31]. In Figure 3b, with the addition of 1 vol.% particles, the grains are significantly refined, particles are distributed at grain boundaries, and the continuous coarse phases are reduced. As shown in Figure 3c, when the particle content is increased to 3 vol.%, the grains are further refined, the number of particles increases, and their distribution becomes more uniform, although partial agglomeration of particles begins to appear at grain boundaries. Figure 3d shows that when the particle content reaches 5 vol.%, severe agglomeration of particles occurs at grain boundaries, and the grains coarsen compared to previous conditions. This is because the excessively high particle content increases the viscosity of the solution during the melt reaction process, hindering particle dispersion. Additionally, the high specific surface energy of the particles [32] and the van der Waals forces between particles [33] lead to the agglomeration of large-sized particles. These agglomerated large-sized particles lose their ability to act as effective heterogeneous nucleation sites. According to classical nucleation theory, the efficiency of heterogeneous nucleation is inversely proportional to the particle size; conversely, excessively large aggregates reduce the nucleation rate, resulting in a diminished number of nucleation sites during solidification. Consequently, grains continue to grow on a limited number of nuclei, ultimately forming coarse primary crystals.

**Figure 4:**
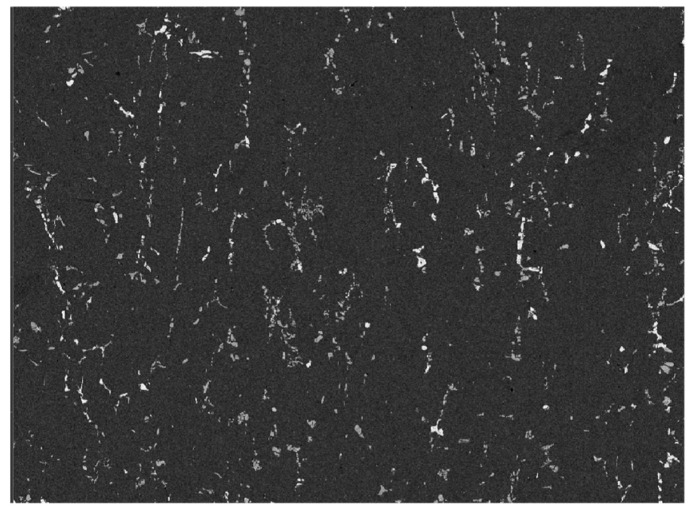
SEM micrographs of (ZrB_2_ + Al_2_O_3_)/7085Al composites after rolling.

Figure 4 shows SEM images of the (ZrB_2_ + Al_2_O_3_)/7085Al composite before and after rolling. As shown in Figure 3c, prior to rolling, particles are predominantly distributed at grain boundaries, with a small number dispersed within grains. In Figure 4, after 80% rolling deformation at 450 °C, particle clusters are crushed and reduced in size, while coarse precipitates become fragmented. The particles are now primarily aligned along the rolling direction, which is attributed to the flow of the matrix during the rolling process.

This section investigated the microstructure of the (ZrB_2_ + Al_2_O_3_)/7085 aluminum matrix composite. The presence of in situ synthesized ZrB_2_ and Al_2_O_3_ nanoparticles was confirmed by XRD and SEM/EDS. As the particle content increased from 0 to 3 vol.%, grain refinement (average size reduced to 40.6 μm) occurred due to heterogeneous nucleation and grain boundary pinning effects. However, excessive particles (5 vol.%) led to abnormal grain coarsening (63.8 μm) caused by agglomeration. After rolling, the particles aligned along the rolling direction, fragmenting coarse precipitates and optimizing their distribution.

### 3.2. Microstructure of (ZrB_2_ + Al_2_O_3_)/7085-Er Mposites

From the above analysis, it can be concluded that excessive particle content leads to severe particle agglomeration and coarsens grains, introducing additional defects. To address these issues caused by particle addition, this study introduces the rare earth element Er to mitigate particle clustering.

**Figure 5:**
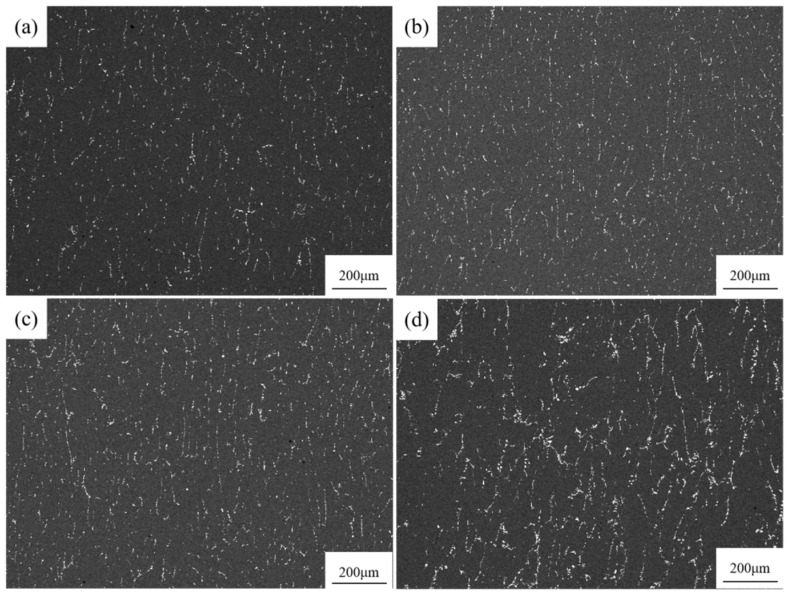
SEM micrographs of 7085Al composites reinforced by Er with different contents after rolling: (**a**) 0 wt.% Er; (**b**) 0.1 wt.% Er; (**c**) 0.3 wt.% Er; (**d**) 0.5 wt.% Er.

Figure 5 presents SEM images of rolled 7085Al composites reinforced with varying Er contents. As shown in Figure 5a, without Er addition, the grains remain relatively coarse. When 0.1 wt.% and 0.3 wt.% Er are added, significant grain refinement is observed. However, at 0.5 wt.% Er, grains become coarser again, and large-sized phases persist even after rolling. This is attributed to the limited solid solubility of rare earth elements in molten aluminum. Excess Er promotes the formation of coarse rare-earth-containing compounds, indicating that higher Er content does not guarantee better performance. Therefore, 0.3 wt.% Er is selected as the optimal concentration to effectively alleviate particle-related issues while avoiding undesirable phase formation.

When 0.1 wt.% and 0.3 wt.% Er are added, significant grain refinement is observed. However, at 0.5 wt.% Er, grains become coarser again, and large-sized phases persist even after rolling. This is attributed to the limited solid solubility of rare earth elements in molten aluminum. Excess Er promotes the formation of coarse rare-earth-containing compounds, indicating that higher Er content does not guarantee better performance. Therefore, 0.3 wt.% Er is selected as the optimal concentration to effectively alleviate particle-related issues while avoiding undesirable phase formation.

**Figure 6:**
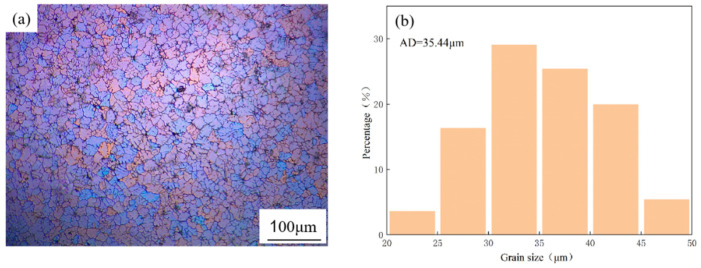
OM pattern and grain size distribution image of 0.3 wt.% Er/3 vol.% (ZrB_2_ + Al_2_O_3_)/7085Al. (**a**) OM pattern; (**b**) grain size distribution image.

Figure 6a shows the polarized light micrograph of the 3 vol.% (ZrB_2_ + Al_2_O_3_)/7085Al composite with 0.3 vol.% Er. Compared to Figure 2c (without Er addition), severe dendritic segregation is observed in the composite without Er, leading to non-uniform grain sizes and numerous defects. After adding Er, the dendritic segregation within grains is significantly alleviated, and the microstructure transitions from dendritic crystals to equiaxed grains with improved uniformity. This refinement occurs because Er forms precipitated phases in the composite, which act as nucleation sites to promote grain formation and refinement. As shown in Figure 6b, the average grain size, calculated using Image-J software, is 35 μm.

**Figure 7:**
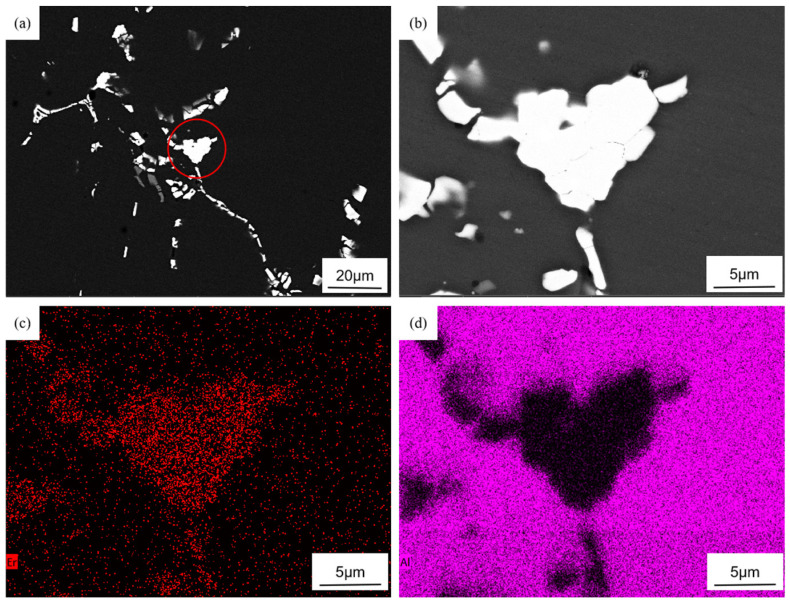
Primary phase containing rare earth Er of 7085Al: (**a**) low magnification; (**b**) high magnification; (**c**) element distribution of Er; (**d**) element distribution of Al.

**Figure 8:**
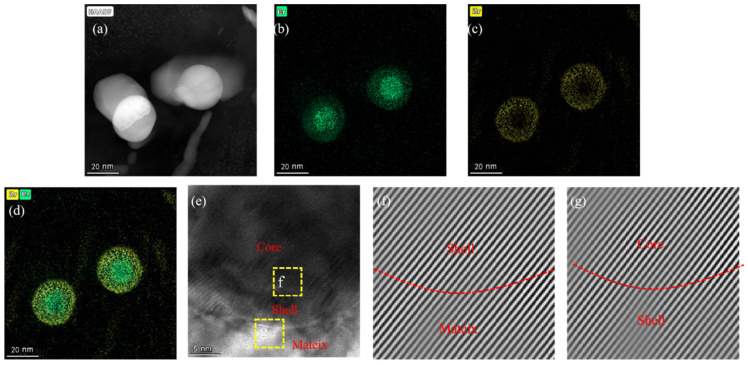
TEM images of Al_3_(Er,Zr) strengthening phase: (**a**) HAADF of Al_3_(Er,Zr) strengthening phase, (**b**) element distribution of Er, (**c**) element distribution of Zr, (**d**) element distribution of (Zr + Er), (**e**–**g**) Inverse Fast Fourier Transform (IFFT) images.

In addition to the enhanced dispersion of particles due to rare-earth addition, the precipitation of rare-earth-containing secondary phases significantly strengthens the aluminum alloy. After adding Er, the primary precipitates formed in the composite are Al_3_Er and Al_3_(Er,Zr). The precipitated phase can form a semi-ferroelectric structure with the matrix. In contrast, the Al_3_(Er,Zr) phase, incorporating the slow-diffusing element Zr, the matrix, exhibits high melting points, excellent stability, and provides effective dispersion strengthening [34]. Al_3_Er, with its cubic crystal structure, serves as a nucleation site for α-Al, thereby refining grains. Since Al_3_Er lacks metastable phases, its equilibrium structure is the L12 type [35,36]. Due to the low solid solubility and rapid diffusion rate of Er in molten aluminum, Al_3_Er typically forms during solidification through the eutectic reaction (L → α-Al + Al_3_Er). Figure 7a shows the backscattered electron (BSE) micrograph of the 0.3 wt.% Er-modified composite. After magnification, Figure 7b clearly displays primary Al_3_Er phases (bright white blocky particles) with diameters of approximately 10 μm. As shown in Figure 7c,d, through EDS analysis performed on Figure 7c, the elements represented by red in the figure are identified as Er, while those indicated by purple correspond to Al, confirming that the white blocky particles shown in the figure are indeed Al_3_Er. Although Al_3_Er has a thermodynamically stable L12 structure, its resistance to coarsening is relatively poor. In contrast, the Al_3_(Er,Zr) phase, incorporating the slow-diffusing element Zr, exhibits improved coarsening resistance. The coherency between the Zr-rich shell and the matrix is superior to that of monolithic Al_3_Er. The core–shell structure of Al_3_(Er,Zr) exhibits a lower Gibbs free energy at elevated temperatures. The elemental concentration gradient between the Er-rich core and Zr-rich shell within this structure further hinders the long-range diffusion of solute atoms under high-temperature conditions. Figure 8a displays the high-angle annular dark-field (HAADF) image of Al_3_(Er,Zr). Heavy elements Er and Zr exhibit bright contrast due to their strong electron scattering capability, while the light element Al appears with dark contrast. As shown in Figure 8b–d, green corresponds to Er, which is concentrated in the central region, while yellow represents Zr, predominantly distributed in the peripheral annular area; since Er diffuses faster than Zr, Al_3_Er precipitates nucleate first, and Zr subsequently enriches around these nuclei, forming a core–shell structured Al_3_(Er,Zr) phase with an Al_3_Er core and an Al_3_Zr shell. Figure 8e illustrates the distribution of the Al_3_(Er,Zr) shell, core, and α-Al matrix. Figure 8f was obtained by applying Inverse Fast Fourier Transform (IFFT) to the shell–matrix region, while Figure 8g was generated by IFFT analysis of the core–shell interface. Both images reveal that atomic arrangements at the shell–matrix and core–shell junctions remain ordered, with no observable lattice distortion. This confirms the absence of lattice mismatch between the α-Al matrix and Al_3_Zr shell, as well as between the Al_3_Zr core and shell. This also confirms the coherent interfaces between the matrix, shell, and core, and demonstrates strong bonding in the Al_3_(Er,Zr) precipitates.

This section explores the optimization effects of the rare earth Er on the microstructure of the composite material. Adding 0.3 wt.% Er alleviates particle agglomeration, eliminates dendritic segregation, and forms uniform equiaxed grains (average 35 μm). Er and Zr synergistically form core–shell structured Al_3_(Er,Zr) precipitates (Er-rich core/Zr-rich shell) with semi-coherent interfaces and excellent thermal stability. Excessive Er (0.5 wt.%) generates coarse rare earth compounds due to low solid solubility, impairing performance.

### 3.3. Tensile Property Testing

**Figure 9:**
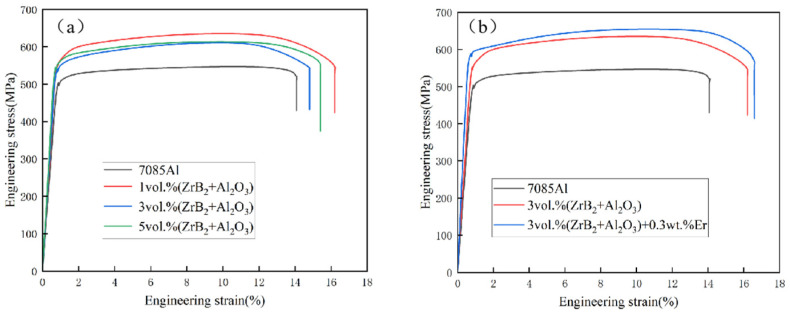
Stress–strain curves of (ZrB_2_ + Al_2_O_3_)/7085 composites: (**a**) (ZrB_2_ + Al_2_O_3_)/7085 composites, (**b**) (ZrB_2_ + Al_2_O_3_)/7085-Er composites.

Figure 9 shows the stress–strain curves of the rolled composite after heat treatment. For the 7085Al alloy without particle addition, the tensile strength is 546.9 MPa with an elongation after fracture of 14.1%. In this case, the absence of strengthening phases results in limited dispersion strengthening and lower resistance to dislocation motion, leading to relatively low strength. When 1 vol.% (ZrB_2_ + Al_2_O_3_) particles are added, the tensile strength increases to 613.8 MPa with the elongation after fracture of 15.4%. This improvement is attributed to dispersion strengthening via the Orowan mechanism, where nanoparticles hinder dislocation motion. Additionally, according to the CTE (Coefficient of Thermal Expansion) strengthening mechanism, the larger mismatch in CTE between ZrB_2_/Al_2_O_3_ and the Al matrix, combined with higher particle content and smaller particle size, leads to higher CTE strengthening. For 3 vol.% (ZrB_2_ + Al_2_O_3_) particles, the tensile strength further rises to 635.4 MPa, representing a 16.2% increase compared to the matrix. with the elongation after fracture reaching 16.2%, representing a 14.9% increase compared to the matrix. The strength of the composite is further enhanced at this stage; the increased particle content enhances both dispersion and CTE strengthening effects. Moreover, the uniform particle distribution (without severe agglomeration) effectively obstructs dislocation movement. However, at 5 vol.% particle addition, the tensile strength drops to 611.3 MPa with the elongation after fracture of 14.8%. This decline is caused by excessive particle agglomeration, forming large clusters and porosity defects. Additionally, weakened interfacial bonding between clusters and the matrix promotes crack initiation (cracks preferentially propagate along interfaces), leading to a reduction in strength. With 3 vol.% particles + 0.3 wt.% Er, the composite achieves a tensile strength of 654.8 MPa, representing a 19.7% increase compared to the matrix, and the elongation is 16.6%, representing a 17.7% increase compared to the matrix. Er refines matrix grains by inhibiting grain growth (via the Hall–Petch effect) and enhances interfacial bonding between particles and the matrix, reducing agglomeration and suppressing crack initiation. The uniform particle distribution and strengthened interfaces, combined with Er addition, optimize the balance between strength and ductility, enabling synergistic enhancement of both properties.

**Figure 10:**
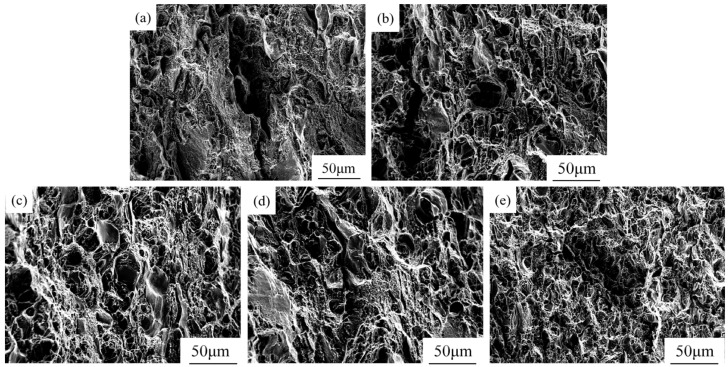
SEM images of fracture surfaces of the (ZrB_2_ + Al_2_O_3_)/7085 composites: (**a**) 7085 alloy, (**b**) 1 vol.% (ZrB_2_ + Al_2_O_3_)/7085 composites, (**c**) 3 vol.% (ZrB_2_ + Al_2_O_3_)/7085 composites, (**d**) 5 vol.% (ZrB_2_ + Al_2_O_3_)/7085 composites, (**e**) 3 vol.% (ZrB_2_ + Al_2_O_3_)/7085-Er composites.

As shown in Figure 10, the 7085 aluminum alloy primarily undergoes brittle fracture. Cracks propagate either transgranularly (through grain interiors) or intergranularly (along grain boundaries). The fracture surface exhibits metallic granular features with a shiny appearance, accompanied by cleavage facets and minimal dimples, indicating dominant brittle behavior. With the addition of 1 vol.% ZrB_2_ + Al_2_O_3_ particles, the composite fracture surface shows sparse, larger-sized dimples alongside residual cleavage facets, suggesting partial ductility improvement but predominantly brittle fracture. When the particle content increases to 3 vol.%, the cleavage facets disappear, and the fracture surface reveals a significant increase in the number of dimples with refined sizes, along with a marked reduction in brittle regions. However, excessive particle addition (5 vol.%) leads to severe particle clustering, exposing large particle clusters on the fracture surface. These clusters act as crack initiation sites due to weakened interfacial bonding, deteriorating mechanical performance. The introduction of rare earth Er further modulates the fracture behavior. Er refines grains and enhances interfacial bonding between particles and the matrix, resulting in smaller, more densely distributed dimples on the fracture surface. This effectively suppresses crack initiation and propagation, improving overall ductility. Additionally, Er mitigates particle clustering at high particle contents, reduces interfacial defects, and optimizes the synergy between strength and toughness.

This section analyzes the variation in mechanical properties. The composite with 3 vol.% in situ nano (ZrB_2_ + Al_2_O_3_) particles and 0.3 wt.% Er achieves optimal mechanical performance after T6 treatment: tensile strength of 654.8 MPa (a 19.7% increase compared to the matrix) and elongation of 16.6% (a 17.7% improvement). The addition of Er effectively suppresses crack initiation and propagation, and the fracture surface exhibits a ductile–brittle mixed fracture mode.

## 4. Discussion

### 4.1. Synergistic Strengthening Mechanisms of Composite Materials

Based on the aforementioned research, the primary strengthening mechanisms in in situ nanocomposites include grain refinement strengthening, Orowan strengthening, and CTE strengthening. The enhancement of the composite material’s mechanical properties is achieved through the combined effects of these strengthening mechanisms.

(1) Grain Refinement Strengthening

Grain refinement strengthening is a mechanism that improves material strength by reducing grain size. When dislocations within grains move near grain boundaries, they are obstructed by the boundaries, leading to dislocation pile-up. To sustain further deformation, external stress must be increased. Moreover, finer grains result in a higher proportion of grain boundaries, creating more obstacles for dislocation motion. Currently, there are two main mechanisms for matrix grain refinement in aluminum matrix composites: (1) the heterogeneous nucleation mechanism at the reinforcement–matrix interface; (2) the thermal exchange mechanism between reinforcements and the matrix alloy. For in situ ZrB_2_, Al_2_O_3_, and Er-reinforced aluminum matrix composites, since the reinforcing phases are chemically synthesized within the melt, they can act as heterogeneous nucleation sites for α-Al phase, thereby inducing refinement. Therefore, the grain refinement strengthening mechanism in these composites is attributed to heterogeneous nucleation of the matrix on reinforcement surfaces. According to the Hall–Petch equation, The grain refinement strengthening increment can be expressed by the following formula:

(2) $$\;∆\sigma_{grain}=\sigma_{0}+\frac{k}{\sqrt{d}-\sqrt{d_{0}}}\;\;$$

In the formula, $\;∆\sigma_{grain}$ represents the grain refinement strengthening contribution, $\sigma_{0}$ is the frictional stress within the grain, *k* is the material constant, *d* is the matrix grain size, and *d*_0_ is the refined grain size. In this equation, *k* and *d* are fixed values. A smaller *d*_0_ (i.e., finer grains) leads to a greater grain refinement strengthening contribution.

(2) Orowan Strengthening

Orowan strengthening is the strengthening effect caused by the obstruction of dislocations passing through closely spaced, fine hard particles. In in situ ZrB_2_, Al_2_O_3_, and Al_3_(Er,Zr) particle-reinforced aluminum matrix composites, due to the fine size and high volume fraction of the particles, most particles are distributed within the grain boundaries. According to Orowan’s dislocation obstruction theory, the smaller the interparticle spacing, the greater the curvature of dislocation lines bypassing the particles, thereby increasing the resistance to dislocation motion and resulting in high material strength. The Orowan strengthening increment can be expressed by the following formula:

(3) $${∆\sigma }_{Orowan}=\frac{0.4\;MGb\ln\frac{\sqrt{2/3}d_{p}}{b}}{\pi \lambda_{P}\sqrt{1-\nu }}\;\;\;\;$$

(4) $$\lambda_{P}={\sqrt{2/3}d}_{p}\left(\sqrt{\pi /4V_{f}}-1\right)$$

In the formula, ${∆\sigma }_{Orowan}$ represents the Orowan strengthening contribution. *M* is the Taylor factor (*M* = 3.07), ν is the Poisson’s ratio (ν= 0.345), *G* is the shear modulus (*G* = 26.2 GPa), *b* is the Burgers vector (*b* = 0.286 nm), *d* is the average size of the reinforcing phase, $\lambda_{P}$ is the average interparticle spacing, *f* is the volume fraction of the reinforcing phase. As indicated by Equations (3) and (4), the smaller the particle size (*d*), the denser the spacing ($\lambda_{P}$), and the higher the volume fraction (*f*), the greater the Orowan strengthening increment (${∆\sigma }_{Orowan}$).

(3) CTE Strengthening

**Figure 11:**
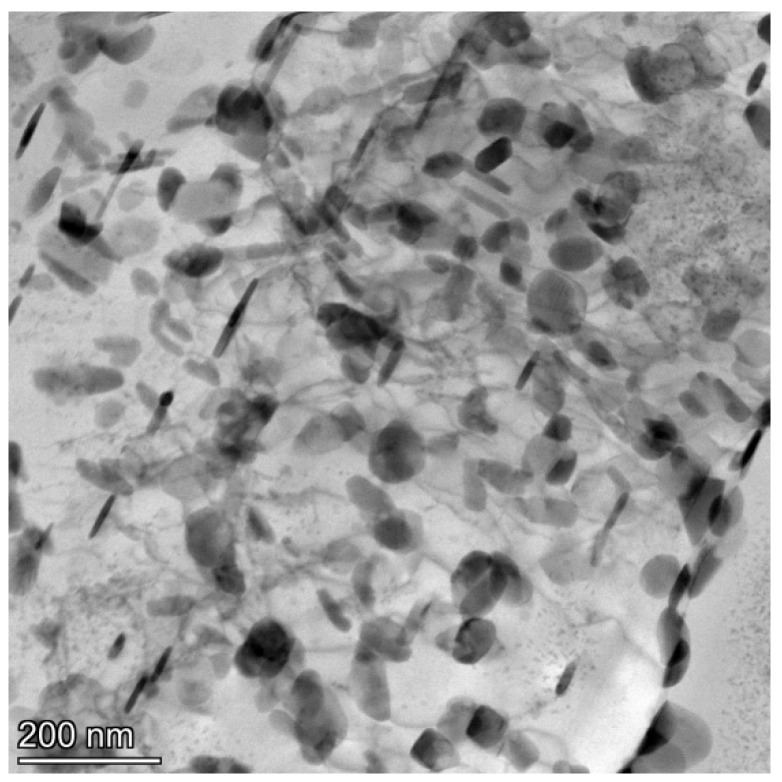
The relationship between in situ nanoparticles and dislocations.

CTE (Coefficient of Thermal Expansion) strengthening is a mechanism that enhances material strength by leveraging the difference in thermal expansion coefficients between the matrix material and the reinforcing phase. When a composite material is cooled from a high temperature to room temperature, thermal mismatch dislocations (as shown in Figure 11) are generated at the interface due to the disparity in thermal expansion coefficients between the matrix and the reinforcing phase. This creates residual stress fields at the interface, which hinder dislocation motion, thereby improving the material’s strength and hardness. The effectiveness of this mechanism depends on the thermal expansion coefficient difference between the matrix and the reinforcing phase, the volume fraction of the reinforcing phase, and the processing parameters of the material. The CTE strengthening increment can be expressed using the following formula:

(5) $$\;∆\sigma_{CTE}=AGb\sqrt{∆\rho }\;$$

(6) $$∆\rho =\frac{2∆\alpha ∆TV_{f}}{bd_{p}\left(1-V_{f}\right)}$$

In the formula, $∆\sigma_{CTE}$ is the CTE strengthening increment, *A* is the constant (*A* = 0.83), ∆ρ is the dislocation density increment, ∆α is the thermal expansion coefficient difference between the matrix and the reinforcing phase, ∆T is the temperature difference between the composite material’s casting temperature and room temperature, and *d* is the average diameter of the nanoparticles. This indicates that a larger thermal expansion coefficient difference (∆α) and a higher volume fraction of the reinforcing phase results in a greater CTE strengthening increment ($∆\sigma_{CTE}$).

From the aforementioned strengthening mechanisms, we can derive the generalized equation describing the relationship between (ZrB_2_ + Al_2_O_3_) nanoparticles and rare earth Er in strengthening the 7085Al alloy:

(7) $${∆}_{a}={∆}_{grain}+{∆}_{Orowan}+{∆}_{CTE}\;$$

Microstructural regulation overcomes the traditional strength–toughness trade-off in aluminum alloys through multi-scale synergistic mechanisms. In fields such as aerospace and rail transportation, the material achieves a significant increase in strength while maintaining excellent fracture toughness, promoting advancements in aerospace technologies toward lightweight design, enhanced reliability, and extended service durability.

### 4.2. Sensitivity Analysis of Process Parameters

Rolling deformation (80% reduction) significantly regulates particle distribution through two mechanisms: (1) fragmentation effect: rolling breaks original clusters (Figure 3c) into submicron particles (Figure 4), forming chain-like distribution along the rolling direction, effectively eliminating dendritic segregation (Figure 6); (2) dynamic recrystallization: heat treatment at 450 °C promotes particle redistribution, but excessive temperatures (>500 °C) may dissolve Al_3_(Er,Zr) precipitates (Figure 7). This aligns with findings by Li et al. [25] in 7075 alloy, suggesting the need to optimize thermomechanical processing windows to balance phase stability and nucleation efficiency.

### 4.3. Potential Industrial Applications

The synergistic reinforcement strategy combining in situ synthesized (ZrB_2_ + Al_2_O_3_) nanoparticles with rare earth Er microalloying demonstrates significant potential for industrial applications requiring high-performance aluminum alloys. This composite material’s exceptional strength–ductility synergy, enhanced thermal stability, and improved interfacial characteristics make it particularly suitable for aerospace structural components such as aircraft wing spars, landing gear assemblies, and spacecraft fuel system parts, where lightweight design and operational reliability are paramount. The material’s demonstrated thermal stability under extreme temperature gradients suggests potential suitability for next-generation hypersonic vehicle skins and rocket engine components operating in high-temperature environments. This inherent heat-resistant characteristic, derived from the nanoparticle-reinforced matrix and optimized interfacial bonding, positions the composite as a candidate material for applications requiring structural reliability in thermally aggressive operating conditions. In automotive engineering, this composite could be applied in safety-critical suspension systems and electric vehicle battery enclosures, benefiting from its damage tolerance and vibration resistance. The manufacturing process’ compatibility with conventional foundry techniques and rolling operations enables cost-effective mass production, while the nanoparticle-reinforced matrix offers extended service life for marine applications requiring corrosion resistance in saltwater environments. The technology’s scalability and performance advantages position it as a competitive solution for advanced transportation systems and renewable energy infrastructure where material efficiency and structural reliability are crucial.

## 5. Conclusions

1. The optimal addition amount of in situ synthesized ZrB_2_ and Al_2_O_3_ nanoparticles is 3 vol.%, which significantly improves both strength and elongation. Excessive addition (5 vol.%) causes particle agglomeration, poor interfacial bonding, and degraded performance.
2. The synergistic coupling of 0.3 wt.% rare earth Er introduces a dual-functionality mechanism: (1) multi-scale particle distribution homogenization through Er-induced Zener pinning effects; (2) the nucleation of Al_3_(Er,Zr) nanoprecipitates further optimizes grains and interfacial bonding. This hierarchical microstructure optimization concurrently achieves grain refinement, interfacial coherence strengthening, and balances strength and toughness.
3. The optimal strength–toughness balance is achieved with 3 vol.% in situ nano (ZrB_2_ + Al_2_O_3_) particles + 0.3 wt.% Er, yielding a tensile strength of 654.8MPa (19.7% increase over the matrix) and elongation after fracture of 16.6% (17.7% improvement compared to the matrix).
4. The synergistic mechanism involves the optimization of nanoparticle content and Er addition, achieving a synergistic enhancement of both strength and toughness in aluminum alloys, providing theoretical support for designing high-performance aluminum matrix composites.
5. Future efforts should focus on exploring consistency control of particle dispersion in industrial-scale fabrication (such as ultrasonic-assisted casting) and stability mechanisms of mechanical properties under complex loading conditions.

## Figures and Tables

**Table 1:** Chemical Compositions (wt.%) of 7085Al Alloy.

Zn	Mg	Cu	Fe	Si	Mn	Cr	Ti	Zr	Al
7.0–8.0	1.2–1.8	1.3–2.0	0.08	0.06	0.04	0.04	0.06	0.08–0.15	Bal

## Data Availability

The original contributions presented in the study are included in the article, further inquiries can be directed to the corresponding author.
